# Free-breathing T2* mapping using respiratory motion corrected averaging

**DOI:** 10.1186/s12968-014-0106-9

**Published:** 2015-01-24

**Authors:** Peter Kellman, Hui Xue, Bruce S Spottiswoode, Christopher M Sandino, Michael S Hansen, Amna Abdel-Gadir, Thomas A Treibel, Stefania Rosmini, Christine Mancini, W Patricia Bandettini, Laura-Ann McGill, Peter Gatehouse, James C Moon, Dudley J Pennell, Andrew E Arai

**Affiliations:** National Heart, Lung, and Blood Institute, National Institutes of Health, DHHS, 10 Center Drive MSC-1061, Bethesda, MD 20892 USA; Siemens Medical Solutions, USA, Inc, Chicago, IL USA; The Heart Hospital, 16-18 Westmoreland Street, London, W1G 8PH UK; Cardiovascular Biomedical Research Unit, Royal Brompton Hospital, Sydney Street, London, SW3 6NP UK

**Keywords:** T2*, R2*, Motion correction, Iron, Mapping, Hemochromatosis, Thalassemia, Cardiovascular magnetic resonance

## Abstract

**Background:**

Pixel-wise T2* maps based on breath-held segmented image acquisition are prone to ghost artifacts in instances of poor breath-holding or cardiac arrhythmia. Single shot imaging is inherently immune to ghost type artifacts. We propose a free-breathing method based on respiratory motion corrected single shot imaging with averaging to improve the signal to noise ratio.

**Methods:**

Images were acquired using a multi-echo gradient recalled echo sequence and T2* maps were calculated at each pixel by exponential fitting. For 40 subjects (2 cohorts), two acquisition protocols were compared: (1) a breath-held, segmented acquisition, and (2) a free-breathing, single-shot multiple repetition respiratory motion corrected average. T2* measurements in the interventricular septum and liver were compared for the 2-methods in all studies with diagnostic image quality.

**Results:**

In cohort 1 (N = 28) with age 51.4 ± 17.6 (m ± SD) including 1 subject with severe myocardial iron overload, there were 8 non-diagnostic breath-held studies due to poor image quality resulting from ghost artifacts caused by respiratory motion or arrhythmias. In cohort 2 (N = 12) with age 30.9 ± 7.5 (m ± SD), including 7 subjects with severe myocardial iron overload and 4 subjects with mild iron overload, a single subject was unable to breath-hold. Free-breathing motion corrected T2* maps were of diagnostic quality in all 40 subjects. T2* measurements were in excellent agreement (In cohort #1, T2*_FB_ = 0.95 x T2*_BH_ + 0.41, r^2^ = 0.93, N = 39 measurements, and in cohort #2, T2*_FB_ = 0.98 x T2*_BH_ + 0.05, r^2^ > 0.99, N = 22 measurements).

**Conclusions:**

A free-breathing approach to T2* mapping is demonstrated to produce consistently good quality maps in the presence of respiratory motion and arrhythmias.

## Background

Breath-held, segmented T2*-measurement is the gold standard method using cardiovascular magnetic resonance (CMR) for diagnosis of diseases involving accumulation of iron in the liver and heart [1-3]. Quantification of T2* is used in guiding therapy and clinical management [3]. Measurement of T2* is typically based on performing an exponential fit to values in a septal region of interest (ROI) in order to improve the signal-to-noise ratio (SNR) and therefore quality of the T2* fit, and where the susceptibility gradient is low thus avoiding intra-voxel dephasing. A number of studies have reported heterogeneity of iron distribution in subjects with iron overload due to Thalassemia [3-5]. The measurement of T2* in a septal ROI has been shown to correlate well with the total iron content [3] and these measurements have proven to have high clinical value. Pixel-wise mapping provides a surrogate measure of the iron distribution [4,5]. Pixel-wise mapping techniques cover the entire field of view and provide a context which may be important in identification of artifacts that might otherwise be less apparent. Pixel-wise mapping is fully automatic and, therefore, reduces the analysis time.

Pixel-wise T2* maps based on breath-held segmented image acquisition tend to be fairly noisy and prone to artifacts due to the relatively long breath-hold durations. We propose a free-breathing method based on respiratory motion corrected single shot imaging with averaging to improve the signal to noise. Single shot imaging is inherently immune to ghost type artifacts. The proposed fitting uses a new automatic truncation method to mitigate noise bias related errors by discarding long echo time measurements based on measured SNR.

## Methods

### Imaging

**Figure 1:**
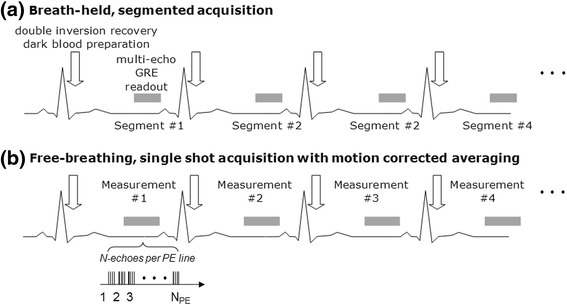
**Multi-echo GRE sequence diagrams for: (a)** **breath-held segmented acquisition, and** **(b)** **free breathing single shot imaging acquired with multiple repetitions and averaged following respiratory motion correction.**

Images were acquired using an investigational prototype [6] multi-echo GRE sequence with gradient flyback for unipolar readout. A dark blood preparation based on double inversion recovery was used to minimize contamination of the myocardium signal by the adjacent blood pool [7-9]. The study compared 2 imaging protocols: (1) a breath-held (BH), segmented acquisition similar to reported T2* protocols [1,9], and (2) a free-breathing (FB), single shot, multiple repetition acquisition with post processing for motion correction (MOCO) and averaging. In cases of severe iron overload, the BH and FB protocols each were acquired with 2 spatial resolutions. The sequences are diagrammed in Figure 1 and protocol parameters are listed in Table 1. Both approaches were ECG triggered to acquire measurements in mid to late diastole. Single RR triggering was used.

**Table 1:** **Imaging protocol parameters**

	**Breath-held, Segmented**		**Free-breathing, MOCO**	
Protocol	BH 256	BH 128	FB 160	FB 128
Readout	Multi-echo GRE (monopolar readout)	Multi-echo GRE (monopolar readout)	Multi-echo GRE (monopolar readout)	Multi-echo GRE (monopolar readout)
Matrix size	256 × 144	128 × 96	160 × 92	128 × 96
FOV (mm^2^) (typical)	360 × 270	360 × 270	360 × 270	360 × 270
Slice thickness (mm)	8	8	8	8
Flip angle (degrees)	18	18	18	18
Parallel imaging acceleration	None	None	4 (using TGRAPPA)	4 (using TGRAPPA)
Bandwidth (Hz/pixel)	977	1953	1078	1953
Echo train length	8	8	8	8
Echo times (ms)	1.6, 3.9, 6.2, 8.5, 10.8, 13.2, 15.5, 17.8	1.1, 2.5, 4.0, 5.4, 6.8, 8.2, 9.6, 11.1	1.2, 3.0, 4.8, 6.7, 8.5, 10.3, 12.1, 14.0	1.0, 2.4, 3.9, 5.3, 6.7, 8.1, 9.5, 11.0
Repetition time (ms)	19.7	12.4	15	11.8
Segments	9	11	23	24
Measurements	1	1	16	16
Total acquisition (heart beats)	17 (including dummy)	10 (including dummy)	16	16

### Processing

**Figure 2:**
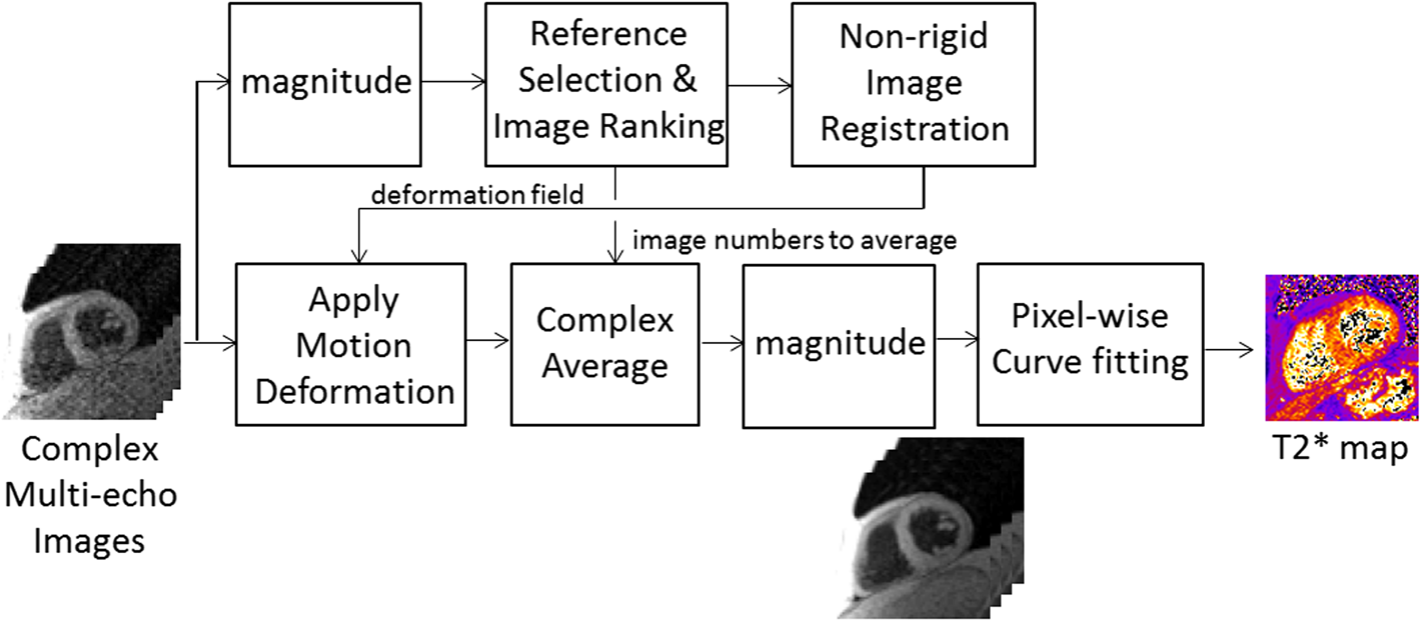
**Processing of free-breathing multiple repetition multi-echo GRE images using respiratory motion correction.**

The T2* was estimated pixel-wise by fitting to a 2-parameter model S_i_ = A exp(−TE_i_/T2*) where A is the signal amplitude, and TE_i_ is the echo time of the i-th image. Fitting used the downhill simplex minimization algorithm proposed by Nelder and Mead [10]. Free-breathing, multiple repetition, single shot acquisitions were motion corrected and averaged (Figure 2). Non-rigid image registration [11] was used to correct in-plane respiratory motion and selective averaging was used, discarding 50% of the images to mitigate through-plane motion. The strategy of discarding data also helps mitigate arrhythmias and poor gating. Selection of a reference image used for image registration used a global metric based on sum of square differences that found the image which agreed best with 50% of the images [12]. This reference image was typically at end expiratory respiratory phase because a large proportion of the respiratory cycle is spent at end expiration. The selection algorithm [12] found the images most similar based on a global sum of square difference with the reference image and selected these images for averaging. Complex averaging was used followed by a root-sum-of-squared magnitude detection to avoid the build-up of noise bias.

Pixel-wise fitting was integrated with the scanner for in-line operation using the Gadgetron framework [13]. For the 8 echo time, 16-repetition protocol, the maps are delivered and displayed within 11 seconds from completion of the scan. The image reconstruction was performed in SNR scaled units [14] such that the standard deviation of the noise was known at the input to the exponential fitting. Automatic truncation of low SNR values was performed to eliminate the noise bias affecting the curve fit for low values of T2*.

### In-vivo studies

The study was divided into 2 parts. The initial cohort of patients was imaged at NIH to demonstrate initial feasibility. In cohort 1, patients (N = 28) included in the study included N = 9 subjects referred specifically for evaluation of iron overload in both the liver and heart; the remainder of this cohort were referred for other known or suspected cardiac disease. Subject were age 51.5 ± 17.6 (mean ± SD) ranging from 20 to 81 (15 male).

Following initial feasibility, a second cohort of patients (N = 12) with known Thalassemia was imaged in London in order to acquire additional data with myocardial iron overload. In cohort 2, subjects were age 30.9 ± 7.5 (m ± SD) ranging from 18 to 42 (7 male). In-vivo data was acquired using 2 imaging protocols to compare the breath-held and free breathing approaches. For subjects with low myocardial T2*, a lower spatial resolution protocol was additionally acquired in order to acquire data at shorter echo times (TE). A single mid-ventricular short axis slice was acquired for each subject. An additional 4-chamber view was acquired in several subjects. This study was approved by the local Institutional Review Boards of the National Heart, Lung, and Blood Institute, Royal Brompton Hospital, and the Heart Hospital, London, UK, and all subjects gave written informed consent to participate.

Imaging was performed on 1.5 T MAGNETOM Aera and MAGNETOM Avanto systems (Siemens Healthcare, Erlangen, Germany), equipped with a 45 mT/m and 200 T/m/s gradient systems and a surface coil array with 32 channels (Avanto) or 30 channels (AERA). For studies performed with the Aera, a local box shim was performed over the heart region to minimize dephasing due to local gradients. Heart rates were measured from the recorded ECG.

### Image analysis

Analysis of image quality was conducted by 2 observers using the following 5 point scale: 1 - Very poor image quality with unusable images; 2 - Average image quality, not all of septum clearly seen and a lot of artifact; 3 - Good image quality with moderate septal artifact; 4 - Very good quality, with minimal septal artifacts; 5 - Excellent image quality with no significant septal artifact. The scoring criteria were adopted from Smith, et al. [8] where the first 2 categories were combined into a single non-diagnostic category. In all cases which were considered diagnostic by both readers (i.e., quality score ≥2), the T2* was measured using a manually drawn ROI in the septum. T2* was similarly measured in the liver for cases in which there was an adequately shimmed liver region in the field-of-view. Septal and liver ROI measurements were evaluated using a scatter plot, and the LV ROI measurements were further compared using Bland Altman analysis. Bland Altman analysis plots the difference in LV ROI measurement of cardiac T2* for the 2 methods versus their means.

## Results

### In-vivo measurements: cohort 1

T2*-maps were acquired with both protocols in all 28 subjects. A single subject with hemochromatosis had severe myocardial iron overload with T2* < 5 ms. T2* for the remaining subjects were considered normal in the heart (>20 ms) [1]. The mean heart rate was 65.7 ± 11.3 bpm (mean ± SD), ranging from 46 to 90 bpm. There were significant arrhythmias noted in N = 4 subjects.

**Figure 3:**
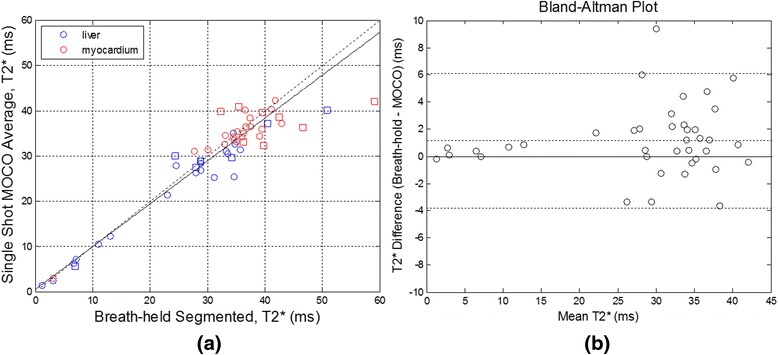
Comparison of T2* measurements in cohort 1: (a) **interventricular septum (n = 28) (red) and liver (n = 26) (blue) ROIs for free-breathing, single shot MOCO vs breath-held segmented protocols.** Studies with non-diagnostic quality segmented breath-hold scans (n = 8 myo and n = 7 liver) are marked with squares. Dotted line represents the identity and solid line represents best fit to all data (r^2^ = 0.93). **(b)** Bland Altman plot of difference and 95% estimated confidence range between breath-held segmented and free-breathing, single shot MOCO protocols for liver and myocardium measurements combined (n = 39, excluding non-diagnostic studies).

The T2* measurements (Figure 3(a)) (N = 20 LV septum and N = 19 liver) excluding the studies which were rated as non-diagnostic had excellent correlation between methods (T2*_FB_ = 0.95 × T2*_BH_ + 0.41, r^2^ = 0.93, N = 39). The liver was not imaged in the short axis slice for 1 subject. Differences between ROI measurements for the LV septum and liver ROIs combined (N = 39) are displayed in a Bland Altman plot (Figure 3(b)) which indicates the mean difference and 95% estimated confidence ranges as dotted lines, showing excellent agreement. Bias was approximately 1 ms. Measurements for images scored as non-diagnostic are shown in Figure 3(a) using square symbol markers.

### In-vivo measurements: cohort 2

**Figure 4:**
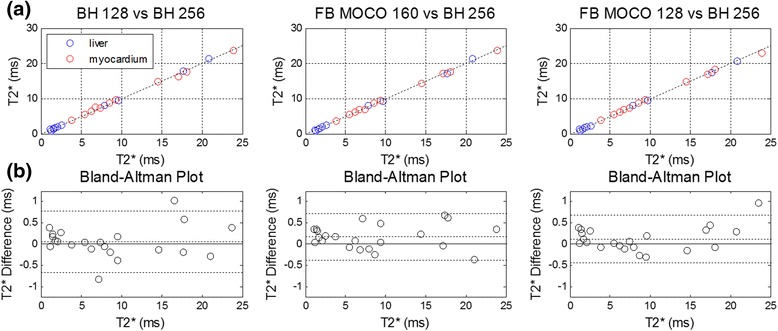
Comparison of T2* measurements in cohort 2: (a) **interventricular septum (n = 11) (red) and liver (n = 11) (blue) ROIs for free-breathing, single shot MOCO vs breath-held segmented protocols.** Dotted line represents the identity and solid line represents best fit to all data (r^2^ > 0.99, all protocols). **(b)** Bland Altman plot of difference and 95% estimated confidence range between breath-held segmented and free-breathing, single shot MOCO protocols for liver and myocardium measurements combined (n = 22).

T2* maps were acquired in 12 subjects of which 11 were of diagnostic quality for both FB and BH method. A single subject was unable to breath-hold. The mean heart rate was 64.2 ± 12.7 bpm (mean ± SD), ranging from 50 to 90 bpm. In this 2nd cohort, myocardial T2* indicated severe myocardial iron overload (<10 ms) in 7 subjects, mild iron overload in 4 subjects (14 ms < T2* < 20 ms), and normal (>20 ms) in 1 subject. T2* maps were acquired using 4 protocols, and the myocardial T2* in a septal ROI was compared for the BH128, FB160, and FB128 protocols against the BH256 protocol. Comparisons including scatter plots and Bland-Altman plots are shown in Figure 4. Bias was less than 0.25 ms for this cohort. Least squares linear fits between methods were T2*_BH128_ = 0.95 × T2*_BH256_ + 0.50 (r^2^ > 0.99, N = 22), T2*_FB160_ = 0.98 × T2*_BH256_ + 0.05 (r^2^ > 0.99, N = 22), and T2*_FB128_ = 0.96 × T2*_BH256_ + 0.38 (r^2^ > 0.99, N = 22) for the 3 comparisons.

### Quality scores

Quality scores were higher for FB than for BH (p < 0.0001, n = 80 aggregating both cohorts and readers). In cohort 1, scores for reader #1 were 2.6 ± 1.3 and 3.6 ± 1.1 (mean ± SD, N = 28) for the breath-held segmented and free-breathing MOCO protocols, respectively. Quality scores for reader #2 were 2.5 ± 1.2 and 3.4 ± 0.7 (mean ± SD, N = 28) for the breath-held segmented and free-breathing MOCO protocols, respectively. Reader #1 found the MOCO method to have an average score that was 1.0 higher than the breath-held method, and reader #2 found the MOCO method to be 0.9 better than breath-held. In cohort 1, reader #1 scored the breath-hold, segmented maps for 6 studies as non-diagnostic, and reader #2 scored 8 studies as non-diagnostic including the same 6 as reader #1. All free breathing studies were judged as diagnostic quality. One of the patients referred for evaluation of iron overload had non-diagnostic quality breath-held T2* maps and was positive for iron overload from the free breathing protocol (T2* = 5.4 ms).

In cohort 2, quality scores for reader #1 were 4.3 ± 1.1 and 4.8 ± 0.5 (mean ± SD, N = 12) for the breath-held segmented and free-breathing MOCO protocols, respectively. Quality scores for reader #2 were 3.8 ± 1.2 and 3.9 ± 0.9 (mean ± SD, N = 12) for the breath-held segmented and free-breathing MOCO protocols, respectively. Reader #1 found the MOCO method to have an average score that was 0.5 higher than the breath-held method, and reader #2 found the MOCO method to be 0.2 better than breath-held. In cohort 2, there was a single study for which the BH maps were deemed to be non-diagnostic by both readers.

### Example T2* maps

**Figure 5:**
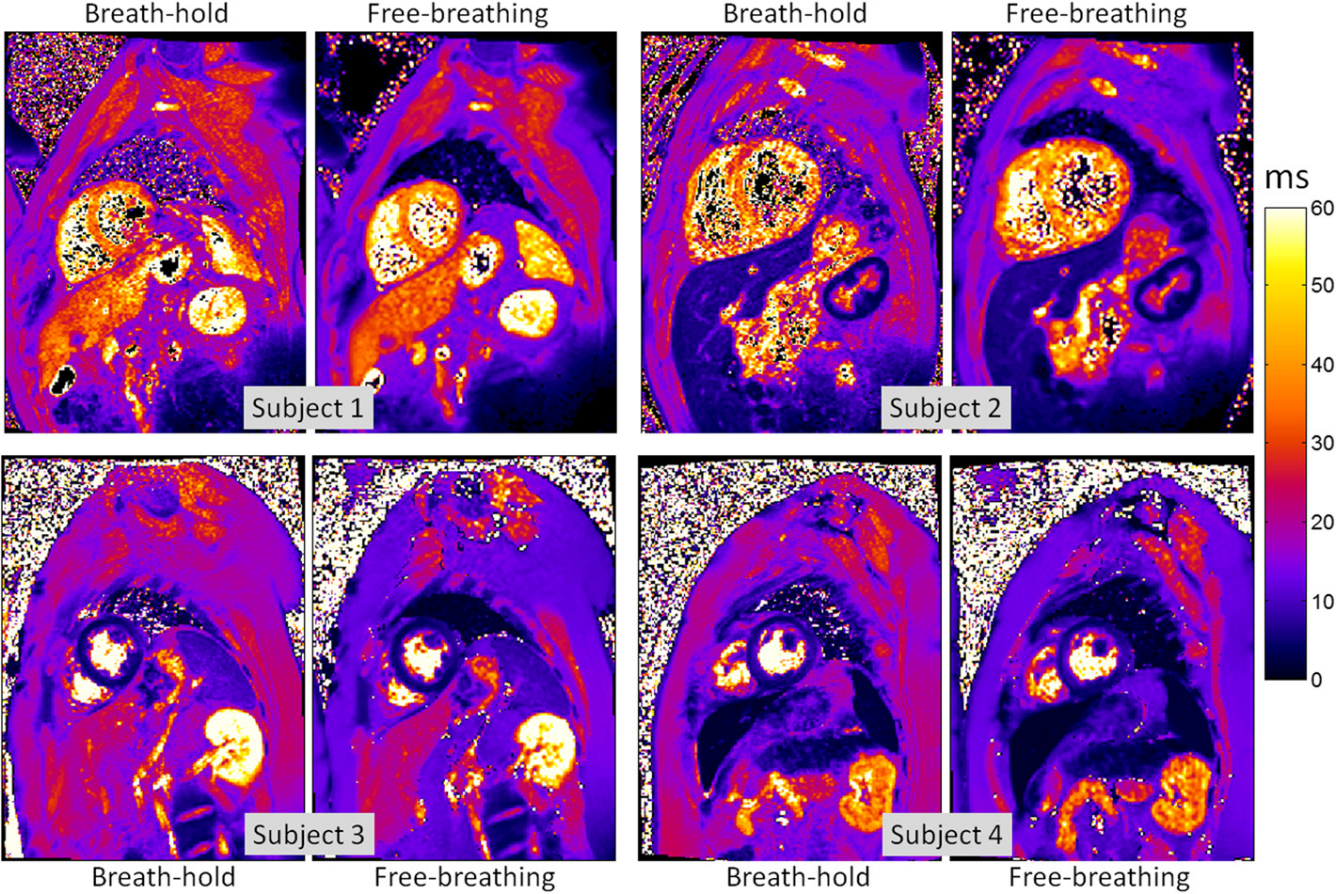
Example of good quality in-vivo T2* maps for 4 subjects comparing breath-held segmented method (left), free-breathing, single shot motion corrected averaging method (right).

**Figure 6:**
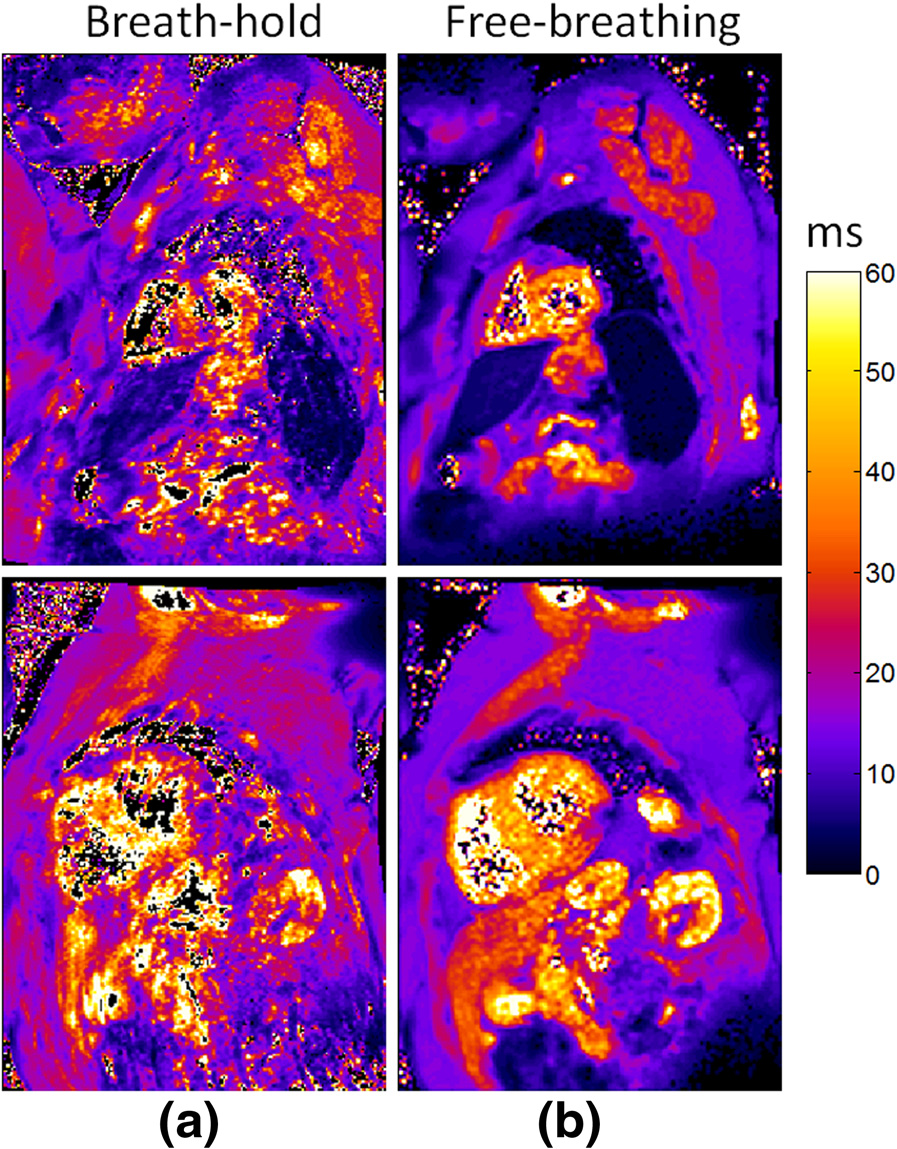
Example of in-vivo T2* maps for subject with normal T2* in the heart in which breath-held method had non-diagnostic quality: a) **breath-held segmented method, and** **b)** **free-breathing, single shot motion corrected averaging method.**

Example T2* maps for both methods are shown (Figure 5) for 4 subjects in which both methods are of good quality. Subject 1 has normal liver (>6.3 ms) and normal myocardium (>20 ms). Subject 2 has normal myocardium and mild iron overload of the liver (<6.3 ms). Subject 3 has severe myocardial iron overload and normal liver and subject 4 has severe iron overload of both the myocardium (<10 ms) and liver (<1.4 ms). Subjects 1 and 2 were from cohort 1 and subjects 3 and 4 were from cohort 2. Figure 6 shows examples of studies (from cohort 1) for which the breath-held segmented method was rated as non-diagnostic due to artifacts. The subject in Figure 6 (top) has mild iron overload in the liver and normal myocardium, and Figure 6 (bottom) has a normal heart and liver.

**Figure 7:**
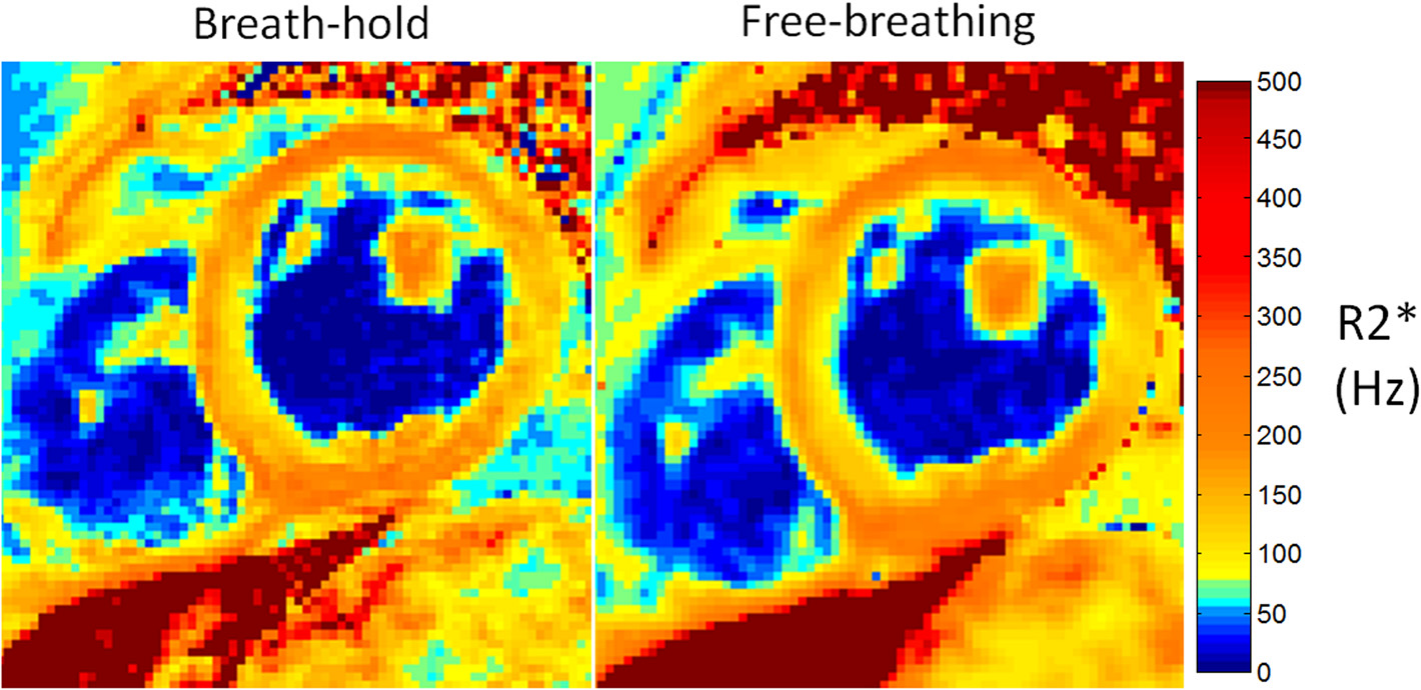
Example R2* maps for subject with severe myocardial iron overload illustrating inhomogeneity of transmural iron distribution in both segmented breath-held (left) and free-breathing, motion corrected averaged (right) protocols.

Heterogeneity of R2* = 1/T2* (iron deposition) was noted in several of the subjects with severe iron overload with a transmural gradient having increased R2* epicardially (Figure 7) consistent with previous reports [3-5]. In all cases, the appearance of the heterogeneity was similar between FB and BH methods. The color map used here distinguishes between normal myocardium (R2* < 50 Hz), mild (50 Hz < R2* < 71 Hz), moderate (71 Hz < R2* < 100Hz), and severe (R2* > 100 Hz). The liver had severe iron overload (R2* ≈ 800 Hz) in this example.

## Discussion

The proposed free-breathing MOCO approach to T2* mapping produces consistently good quality maps in the presence of respiratory motion and arrhythmias. MOCO T2* maps have fewer artifacts than current breath-hold segmented protocols and measurements of T2* are in excellent agreement with the breath-hold segmented protocols in subjects who are capable of breath-holding. In this study, the cohort 1 population was older, less capable of breath-holding, and had a number of instances of significant arrhythmias, compared to the cohort 2 which was a smaller group of younger age. Patients in cohort 2 that were invited to take part in this research study were generally more cooperative and capable of breath-holding than expected in routine clinical practice.

The protocol chosen for breath-held segmented T2* imaging in the heart was the protocol in use by our institution and similar to reported protocols [1,9]. A number of parameter modifications can be made to reduce the breath-hold duration and thereby reduce the instances of non-diagnostic scans. The breath-hold duration of the segmented protocol could be reduced by 40-50%, improving the percentage of diagnostic studies but not entirely mitigating the artifact problem for subjects unable to breath-hold and/or with large variation in RR interval. It is also possible to repeat poor quality breath-hold scans provided that they are evaluated as non-diagnostic at the time of scanning. Parallel imaging could be used in the segmented protocol as done in the single shot method; however this approach was not attractive due to the loss in SNR since the segmented scan has no averaging. Finally, the segmented acquisition is highly sensitive to timing and temporal resolution and often results in ghost artifacts due to cardiac motion, so increasing the number of segments isn’t necessarily an effective means of reducing scan time.

The MOCO scans used 8 averages (of 16 repetitions) which improved the SNR thereby mitigating the parallel imaging loss. The complex averaging approach prevents a build-up of noise bias with increasing averaging, so the number of images acquired and averaged using the free-breathing MOCO approach can be increased to further improve the quality of maps. The poor temporal resolution of 345 ms (scan window) of the single shot method does not result in ghosting but rather a blurring or loss of spatial resolution. Despite the temporal resolution, the MOCO method was highly robust (diagnostic quality in all free breathing scans) and provided high image quality maps.

## Conclusions

A free-breathing approach to T2* mapping is demonstrated to produce consistently good quality maps in the presence of respiratory motion and arrhythmias and with a similar total scan time as existing breath-hold T2* measurement techniques. The approach is fully automatic including the truncation of long echo time measurements at low SNR to mitigate noise bias.

## Abbreviations

BH: Breath-held
FB: Free-breathing
GRE: Gradient recalled echo
MOCO: Motion correction
ROI: Region-of-interest
SD: Standard deviation
SNR: Signal-to-noise ratio
